# Rapid Progression of Angioimmunoblastic T Cell Lymphoma Following BNT162b2 mRNA Vaccine Booster Shot: A Case Report

**DOI:** 10.3389/fmed.2021.798095

**Published:** 2021-11-25

**Authors:** Serge Goldman, Dominique Bron, Thomas Tousseyn, Irina Vierasu, Laurent Dewispelaere, Pierre Heimann, Elie Cogan, Michel Goldman

**Affiliations:** ^1^Department of Nuclear Medicine, Erasme Hospital, Université Libre de Bruxelles, Brussels, Belgium; ^2^Department of Hematology, Jules Bordet Institute, Université Libre de Bruxelles, Brussels, Belgium; ^3^Department of Pathology, UZ Leuven Hospitals, Leuven, Belgium; ^4^Laboratory of Hematology, LHUB, Université Libre de Bruxelles, Brussels, Belgium; ^5^Department of Internal Medicine, CHIREC Hospital, Brussels, Belgium; ^6^I3h Institute, Université Libre de Bruxelles, Brussels, Belgium

**Keywords:** mRNA vaccine, T cell, lymphoma, COVID-19, angioimmunoblastic, follicular

## Abstract

Since nucleoside-modified mRNA vaccines strongly activate T follicular helper cells, it is important to explore the possible impact of approved SARS-CoV-2 mRNA vaccines on neoplasms affecting this cell type. Herein, we report and discuss unexpected rapid progression of lymphomatous lesions after administration of a BNT162b2 mRNA vaccine booster in a man recently diagnosed with AITL.

## Introduction

The remarkable efficiency of nucleoside-modified SARS-CoV-2 mRNA vaccines has been related to their ability to induce a potent stimulation of T follicular helper (TFH) cells, resulting in persistent germinal center B cell responses (1, 2). Clinically, this might translate into reactive lymphoadenopathy which sometimes may raise a differential diagnosis with a lymphoproliferative disorder (3, 4). At the same time, the possible impact of SARS-CoV-2 mRNA vaccination on pre-existing peripheral T cell lymphoma is still to be determined.

## Case Report

A 66-year-old man with no significant medical history except for hypertension, hypercholesterolemia and type 2 diabetes presented on September 1, 2021 with cervical lymphadenopathies that became recently apparent during a flu-like syndrome. The two doses of BNT162b2 mRNA vaccine had been administered, respectively, 5 and 6 months earlier in the left deltoid. Besides moderate asthenia, he did not report any constitutional symptom. Blood examination indicated a mild inflammatory syndrome, without anemia or white blood cell changes; Lymphocytes immunophenotyping was unremarkable. Protein electrophoresis and immunoglobulin levels were normal and Coombs test was negative.

**Figure 1:**
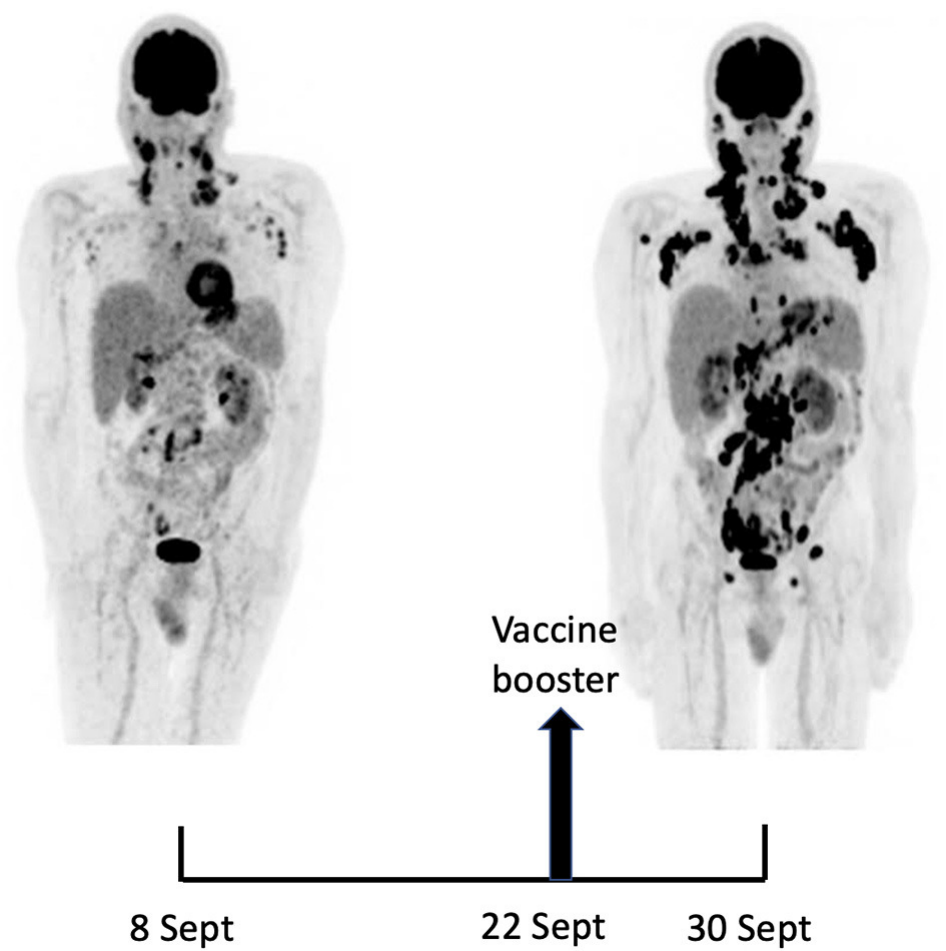
Maximum-intensity-projection images of ^18^F-FDG PET/CT at baseline (8 Sept) and 22 days later (30 Sept), 8 days after BNT162b2 mRNA vaccine injection in right deltoid. 8 Sept: hypermetabolic lymph nodes mainly in the supra-clavicular, cervical, and left axillary regions; restricted gastro-intestinal hypermetabolic lesions. 30 Sept: Dramatic increase in nodal and gastro-intestinal hypermetabolic lesions. Asymmetrical metabolic progression in the cervical, supra-clavicular and axillary area, more pronounced on the right side.

**Figure 2:**
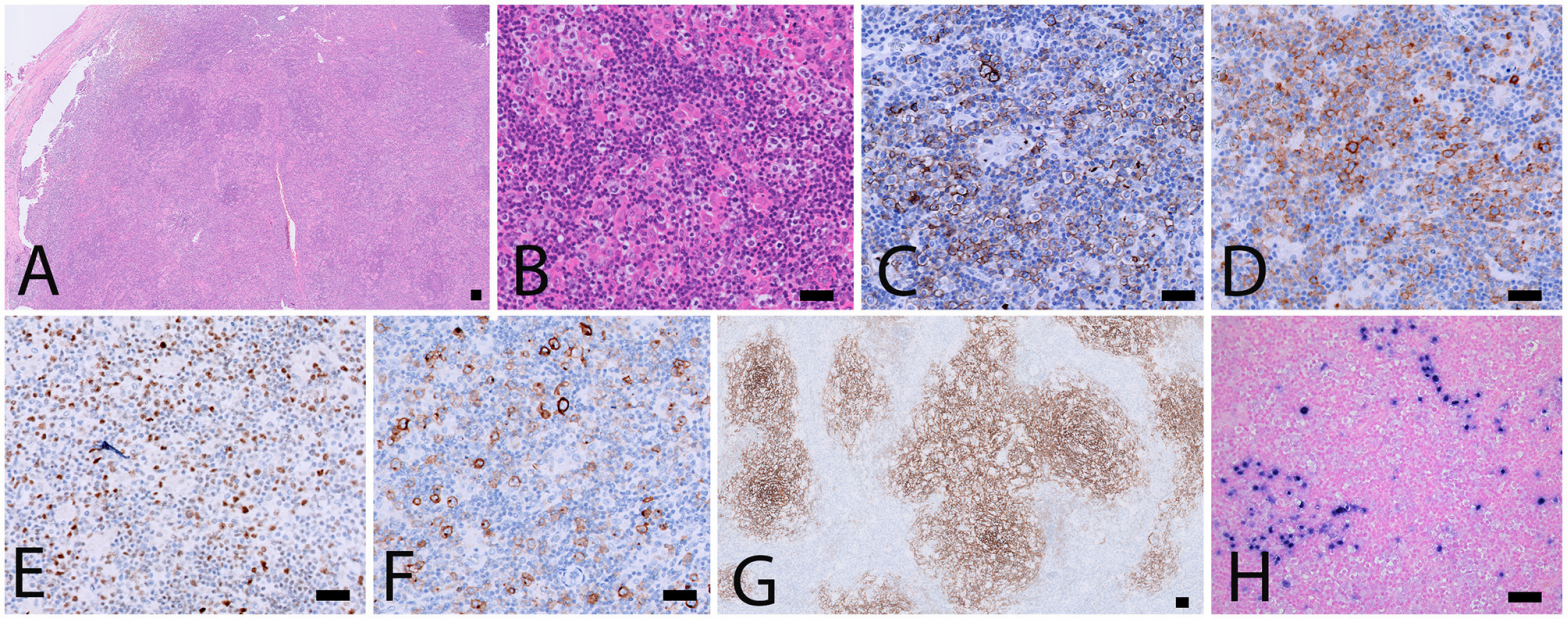
Biopsy specimen. **(A,B)** H&E stainings showing architectural disturbance due to a medium-sized lymphoid population with a clear cell morphology. **(C–F)** Immunohistochemical stainings establishing the TFH origin of the abnormal cell population: CD3+, CD4+, CD10+ (not shown), ICOS+ **(C)**, PD1 **(D)**, BCL6 **(E)** and expression of CD30 **(F)**. CD21 staining **(G)** shows an extended network of follicular dendritic cells. **(H)** Intermediate sized EBV+ immunoblasts by EBER *in situ* hybridization. Scalebar: 100 μm.

A 18F-FDG PET/CT revealed multiple voluminous hypermetabolic lymphadenopathies above and below the diaphragm as well as several extra-nodal hypermetabolic lesions (Figure 1, left panel). Considering a presumptive diagnosis of stage IV lymphoma, a left cervical lymph node biopsy was performed. Pathological examination revealed residual atrophic germinal centers, surrounded by an expanded paracortical area composed of an atypical T-cell infiltrate with clear cell morphology, expressing TFH cell markers (CD3, CD4, PD1, ICOS, BCL6, CXCL13) and a loss of CD7. The paracortical area contained an increased number of high-endothelial venules, supported by an increased number of follicular dendritic cell networks, with some foci of EBV+ B-cell immunoblastic proliferation in the background (Figure 2). These features highly suggested a diagnosis of AngioImmunoblastic T cell Lymphoma (AITL), pattern 2. Next generation sequencing (NGS) performed on the biopsy specimen identified the RHOA G17V mutation characteristic of AITL (5) together with the DNMT3A, IDH2 and TET2 mutations. A TCR-gamma gene rearrangement confirmed a clonal T cell proliferation. Altogether, these findings unambiguously established the diagnosis of AITL. A bone marrow biopsy did not reveal neither morphological nor phenotypic abnormalities, but NGS revealed DMNT3A and TET2 mutations in bone marrow cells with allele frequencies of 41% and 36%, respectively.

Fourteen days after the PET/CT, a booster dose of the BNT162b2 mRNA vaccine was administered in the right deltoid in preparation of the first cycle of chemotherapy. Within a few days following the vaccine booster, the patient reported noticeable swelling of right cervical lymph nodes. In order to get a baseline close to the initiation of the therapy, a second 18F-FDG PET/CT was performed 8 days after the vaccine booster administration, i.e. 22 days after the first one.

It demonstrated a clear increase in number, size and metabolic activity of pre-existing lymphadenopathies at the supra- and sub-diaphragmatic level. Furthermore, new hypermetabolic lymphadenopathies and new hypermetabolic sites had developed since the first examination, in several different locations (Figure 1, right panel). Total lesion glycolysis (TLG) index was used to assess the changes in lymph node activities (6). As compared with the initial test, there was a marked 5.3-fold increase in whole-body TLG, with the increase in the post-booster test being twice higher in the right axillary region than in the left one. In parallel, a mild increase in blood levels of ferritin, C-reactive protein and LDH were noted.

Methylprednisolone administration was initiated immediately after the 2nd PET/CT, followed by a first course of brentuximab vendotin combined with cyclophosphamide, doxorubicin (BV-CHP) according to a recently published protocol (7). At the time of this report, 2 weeks after start of the treatment, clinical examination indicates significant decreased swelling of cervical and axillary lymph nodes, and the overall performance status of the patient is improving. Importantly, comparison of anti-SARS-CoV-2 antibody levels immediately before and 21 days after the vaccine booster did not show a significant change in the production of anti-spike antibodies (171 vs.147 binding antibody units/ml).

## Discussion

Soon after the initiation of the anti-SARS-CoV-2 vaccination campaigns, it appeared that the injection of mRNA vaccines may induce swelling of lymph nodes draining the injection site. Although considered as benign, this vaccine reaction sometimes complicated the interpretation of 18F-FDG PET/CT imaging for suspicion of a neoplastic process affecting lymph nodes (3). When a lymph node biopsy was performed to exclude a malignant process, the pathological picture showed reactive benign changes with prominent germinal centers (3, 8). The differential diagnosis with lymphoma was occasionally complicated by the development of hypermetabolic sites at distance of the injection site, including contralateral lymph nodes or spleen (9, 10). In a patient with mantle lymphoma, PET/CT was suggestive of a relapse but was eventually excluded (11).

Published studies on hypermetabolic lymphadenopathy after SARS-CoV-2 vaccination were recently reviewed and the subject of a meta-analysis (8, 12). Most observations were reported after injection of approved nucleoside-modified mRNA vaccines, namely BNT162b2 (Pfizer-BioNTech) or mRNA-173 (Moderna) (8). Nevertheless, hypermetabolic lymphadenopathies were also observed in 31 health workers following injection of the adenovirus-vectored Vaxveria vaccine (13).

Considering oncologic patients, the most informative study was conducted in a series of 728 patients having received the BNT162b2 mRNA vaccine (14). PET/CT revealed hypermetabolic lymph nodes in the axillary and supraclavicular regions draining the vaccine injection site in 36% of the subjects having received the first dose and 54% of those studied after the 2nd dose. The hypermetabolic lymph nodes were enlarged in 7% of 1st dose vaccinees and 18% of 2nd dose vaccinees. Both differences were statistically significant, demonstrating that the impact on draining lymph nodes was greater after the booster dose, confirming data from the meta-analysis above (12). Regarding the relationship with the underlying malignancy, hypermetabolic lymph nodes were considered as malignant in 5% of the patients while no conclusion regarding the malignant nature could be drawn in 15% of the vaccinees including 16 patients with lymphoma. Interestingly, in none of these studies, the possibility that the mRNA vaccines could have played a role in the development of malignant lymph nodes was considered. Indeed, the consensus so far is that the occurrence of hypermetabolic lymphadenopathies should not question the safety of mRNA vaccines, neither in healthy individuals nor in patients with neoplastic conditions (15).

To the best of our knowledge, this is the first observation suggesting that administration of a SARS-CoV-2 vaccine might induce AITL progression. Several arguments support this possibility. First, the dramatic speed and magnitude of the progression manifested on two 18F-FDG PET-CT performed 22 days apart. Such a rapid evolution would be highly unexpected in the natural course in the disease. Since mRNA vaccination is known to induce enlargement and hypermetabolic activity of draining lymph nodes, it is reasonable to postulate that it was the trigger of the changes observed. Indeed, the increase in size and metabolic activity was higher in axillary lymph nodes draining the site of vaccine injection as compared to their contralateral counterparts. However, pre-existing lymphomatous nodes were also clearly enhanced as compared to the first test. Moreover, new hypermetabolic lesions most likely of lymphomatous nature clearly appeared at distance of the injection site.

In fact, the supposed enhancing action of the vaccine on AITL neoplastic cells is fully consistent with previous observations identifying TFH cells within germinal centers as key targets of nucleoside-modified mRNA vaccines both in animals and in man (1, 2). Malignant TFH cells, the hallmark of AITH, might be especially sensitive to mRNA vaccines when they harbor the RHOA G17V mutation which was present in our case. Indeed, this mutation facilitates proliferation and activation of several signaling pathways in TFH cells (16). Furthermore, mice genetically engineered to reproduce the RHOA G17V and TET2 mutations—both were present in our case—develop lymphoma upon immunization with sheep red blood cells (16). This experimental observation is relevant to RNA vaccines as RNA of sheep red blood cells was shown to be responsible for their ability to stimulate TFH and induce germinal center reaction (17).

Our case first raises the question of the COVID-19 prevention strategy to be used in this patient which is currently poorly protected against COVID-19. On the short term, the only option is to recommend strict masking and social distancing, and to offer him anti-SARS-CoV-2 antibody therapy in case of high-risk contact (16). On the longer term, the use of mRNA vaccines should clearly be avoided while other types of vaccines might be considered.

At this time, extrapolation of the findings of this case to other patients with AITL or other peripheral T cell lymphoma involving TFH cells is premature. AITL patients are rare and their mutation profile is heterogeneous. Furthermore, their immune reactions might be affected by their treatment. It is therefore unlikely that existing pharmacovigilance systems will be efficient to identify extremely rare cases like ours. Prospective studies involving systematic PET/CT imaging after SARS-CoV-2 vaccination in AITL patients with specified mutation profiles might eventually be needed. Whatever the result of such studies, it should not affect the overall favorable benefit-risk ratio of these much-needed vaccines.

## Conclusion

This observation, which has been posted as a pre-print on the SSRN platform (18), suggests that vaccination with the BNT162b2 mRNA vaccine might induce rapid progression of AITL. Dedicated studies are needed to determine whether this case can be extrapolated to populations of patients with AITL or other peripheral T cell lymphoma involving TFH cells.

### Patient Perspective

The patient is the corresponding author of this case report. He hopes that this report will incentivize investigations to clarify the possible impact of anti-SARS-CoV-2 mRNA vaccination on the course of AITL. He remains convinced that mRNA vaccines represent very efficient products with a favorable benefit-risk ratio.

## Data Availability Statement

The original contributions presented in the study are included in the article/supplementary material, further inquiries can be directed to the corresponding author/s.

## Ethics Statement

Ethical approval was not provided for this study on human participants because the retrospective analysis of the PET/CT data has been approved by the Ethics Committee of the Hôpital Erasme (ref. P2017/020). Being one of the authors, the patient consented to the publication. The patients/participants provided their written informed consent to participate in this study. Written informed consent was obtained from the individual(s) for the publication of any potentially identifiable images or data included in this article.

## Author Contributions

All authors listed have made a substantial, direct, and intellectual contribution to the work and approved it for publication.

## Funding

The department of nuclear medicine of Hôpital Erasme is financially supported by the Fonds Erasme and the Association Vinçotte Nuclear (Belgium).

## Conflict of Interest

The authors declare that the research was conducted in the absence of any commercial or financial relationships that could be construed as a potential conflict of interest.

## Publisher's Note

All claims expressed in this article are solely those of the authors and do not necessarily represent those of their affiliated organizations, or those of the publisher, the editors and the reviewers. Any product that may be evaluated in this article, or claim that may be made by its manufacturer, is not guaranteed or endorsed by the publisher.
